# A Method for Measuring Contact Points in Human–Object Interaction Utilizing Infrared Cameras

**DOI:** 10.3389/frobt.2021.800131

**Published:** 2022-02-14

**Authors:** Jussi Hakala, Jukka Häkkinen

**Affiliations:** Department of Psychology and Logopedics, Faculty of Medicine, University of Helsinki, Helsinki, Finland

**Keywords:** grasping, touch, infrared camera, contact point, prehension movements

## Abstract

This article presents a novel method for measuring contact points in human–object interaction. Research in multiple prehension-related fields, e.g., action planning, affordance, motor function, ergonomics, and robotic grasping, benefits from accurate and precise measurements of contact points between a subject’s hands and objects. During interaction, the subject’s hands occlude the contact points, which poses a major challenge for direct optical measurement methods. Our method solves the occlusion problem by exploiting thermal energy transfer from the subject’s hand to the object surface during interaction. After the interaction, we measure the heat emitted by the object surface with four high-resolution infrared cameras surrounding the object. A computer-vision algorithm detects the areas in the infrared images where the subject’s fingers have touched the object. A structured light 3D scanner produces a point cloud of the scene, which enables the localization of the object in relation to the infrared cameras. We then use the localization result to project the detected contact points from the infrared camera images to the surface of the 3D model of the object. Data collection with this method is fast, unobtrusive, contactless, markerless, and automated. The method enables accurate measurement of contact points in non-trivially complex objects. Furthermore, the method is extendable to measuring surface contact areas, or patches, instead of contact points. In this article, we present the method and sample grasp measurement results with publicly available objects.

## Introduction

Our hands are excellent tools for manipulating objects, and we touch and grasp countless objects every day. Prehension movements are divided into three components: moving the hand to the target, setting finger posture for grasping, and aligning the hand so that grasping is possible (Fan et al., 2006). They enable us to select contact points in the object that allow a stable grip and object manipulation (Zatsiorsky and Latash, 2008; Kleinholdermann et al., 2013).

Prehension movements are guided the visual information about the size, shape and density of the object (Cesari and Newell, 1999). This information is used in preliminary planning of hand movement and grasp properties, but also changes that occur during the hand movement are also taken into account (Bridgeman et al., 1979).

While the operations are easy and effortless for humans, they are not easy for robots. Grasping and manipulating previously unseen objects and operating in unstructured, cluttered, and variable environments has proven to be a very difficult task for robots (Cui and Trinkle, 2021). Although great efforts have been focused on the problem in recent years, it has not been solved (Kleeberger et al., 2020).

There are several ways to approach the problem. One approach is called analytic, in which the grasp is determined by analyzing the shape of the object. This could be done, for example, by analyzing the appearance of objects to determine their grasping affordances (Song et al., 2015; Do et al., 2018).

A second approach is called data-driven, as a dataset is formed to train the robot hand with suitable method, such as deep learning. Examples of the data-driven approach are, for example, a study by Gabellieri et al. (2020), which describe human grasp data collection, where a human operator uses the robot hand to grasp objects and resulting the grasp data is collected with depth cameras and motion tracker. Edmonds et al. (2019) used tactile gloves with force sensors to capture the poses and forces that humans used when opening medicine bottles with safety mechanisms. The data was used to teach a robotic system do conduct the same task.

As grasp data collection is time consuming and expensive, the data has been collected in to databases such as the Columbia Grasp Database, which is based on the GraspIt! Toolkit, and can be used by the research community to develop suitable training methods (Goldfeder et al., 2009).

The critical issue with the data-driven approach is the quality of data that is collected. Human hand trajectory and grasp can be measured in many ways. For example, the interaction can be video-recorded and the interactions manually coded from videos (Cesari and Newell, 1999; Choi and Mark, 2004). However, this is very time consuming and can lead to issues with inter-coder reliability (Kita et al., 1998).

Instead of manual annotation, a computer vision system can detect and track hands from RGB images (Siddharth et al., 2016; Cai et al., 2017; Lyubanenko et al., 2017) or from depth camera images (Oikonomidis et al., 2011). Many of these models perform well in laboratory conditions, but have problems with unconstrained real-life conditions, as large variations in the scenery makes feature extraction difficult (Yang et al., 2015). Attaching visual markers to the hand and tracking the markers (Gentilucci et al., 1992) improves the tracking result, at the cost of making the experimental setup more complex and less natural. However, the most critical problem is hand occlusion, which makes the detection of contact points difficult (Yang et al., 2015; Sridhar et al., 2016) for both manual and computer vision-based methods.

Another category of methods relies on sensors attached to the hand. The sensors in these systems can be electromagnetic (trakSTAR, Ascension Technology Corp., Shelburne, VT, United States; e.g., Chen and Saunders (2018), FASTRAK, Polhemus Corp., Colchester, VT, United States; e.g., Bock and Jüngling (1999), resistive (CyberGlove, Virtual Technologies, Palo Alto, CA, United States; e.g., Ansuini et al., 2007), or infrared (Optotrak, Northern Digital Inc., Waterloo, Ontario, Canada; e.g., Kleinholdermann et al. (2013) and Cressman et al. (2007). Sensors provide accurate hand and joint motion data, but they make the experimental setup less natural and might disrupt hand-object interactions, as both sensors and the related wires may limit movements and change grasping strategies.

As all the current methods have limitations, we introduce a contact point measuring method to overcome these limitations. The criteria during the development work were that the method 1) allows for fast data collection without manual annotation; 2) can measure everyday objects with high ecological validity, i.e., without altering their visual appearance or interfering with interaction; and 3) produces accurate data.

Our method is based on thermal imaging, which has been widely used in medicine (Gizińska et al., 2021; Martinez-Jimenez et al., 2021), surveillance (Ivašić-Kos et al., 2019; Duan et al., 2021), and quality control (ElMasry et al., 2020; Khera et al., 2020). The starting point of our application is the temperature of fingers, which is 29.1 ± 0.6°C at the environmental temperature of 25.4 ± 0.4°C, (Shilco et al., 2019), and which transfers to objects that have been touched. In other words, when a participant touches an object and takes their hand away, there is a short-term heat signature corresponding to the area touched. This heat signature can be used to deduce the grasp type used during the human-object interaction. Furthermore, the heat signature can be used to differentiate between grasp types that look visually similar when the hand is viewed from above, but are actually different as the details of the grip are occluded by the hand.

In the following article we will outline the technical details of the method as well as presenting preliminary data recorded with the system while grasping the YCB object set (Calli et al., 2015).

## Methods

**FIGURE 1:**
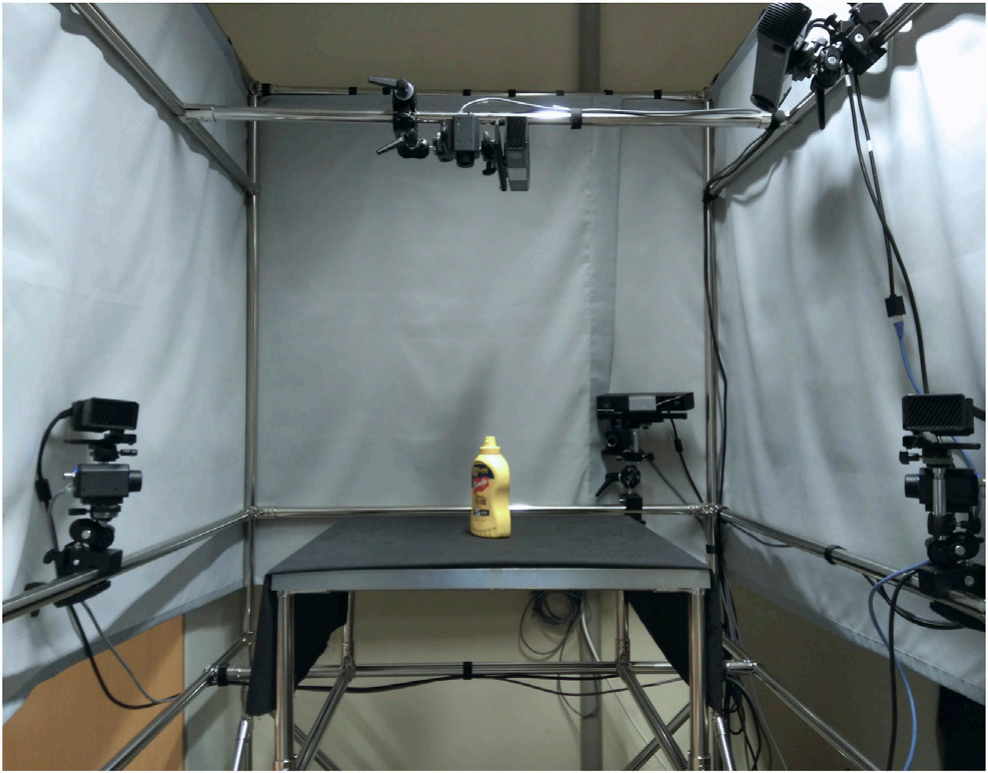
The measuring station with a target object.

The measuring station (Figure 1) consists of four FLIR A65 infrared cameras (FLIR Systems, Wilsonville, OR, United States), one PhoXi 3D Scanner M structured light scanner (Photoneo s.r.o., Bratislava, Slovakia), a control computer, and a frame structure with a flat surface in the middle for placing the target object. For future development, we also included four Xbox One Kinect Sensors (Microsoft Corporation, Redmond, WA, United States) in the configuration.

**FIGURE 2:**
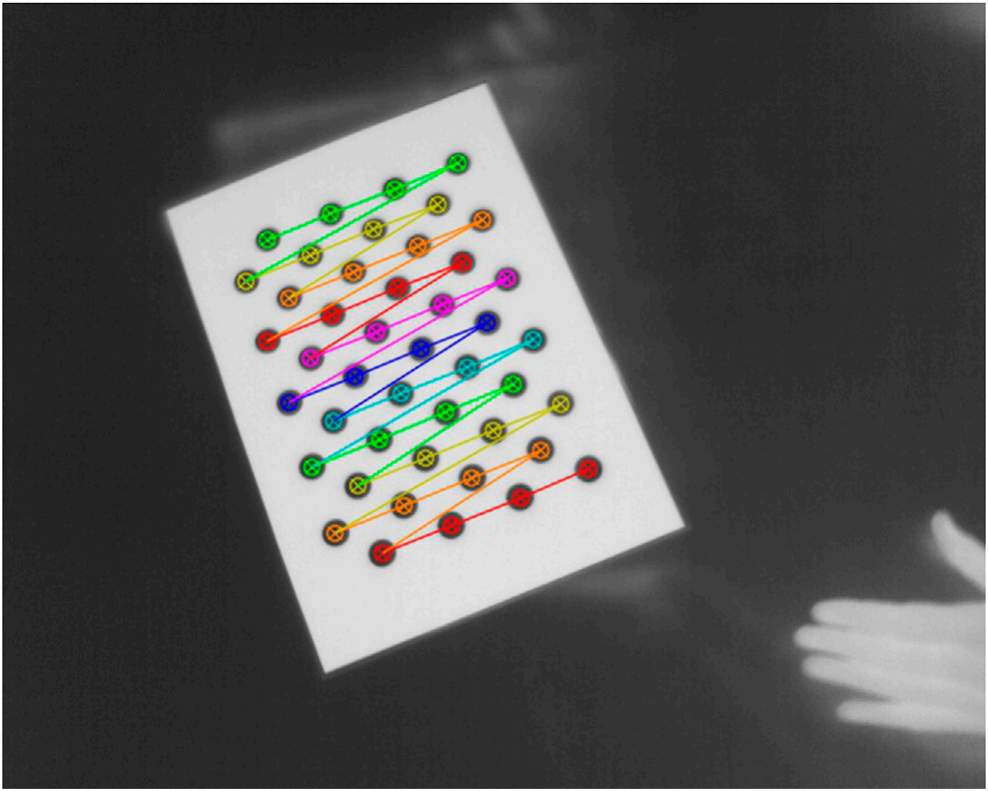
The calibration pattern detected in the infrared image. The warm PVC coated area appears white in the image, whereas the uncoated metal circles reflect the colder surrounding environment.

Calibration of the camera system requires a target where the pattern features are detectable in images recorded with all the cameras in the system. The system consists of four types of cameras that record different bands of the spectrum. The infrared cameras record wavelengths 7.5–13.0 µm (Flir systems, 2016). The 3D scanner utilizes a 638 nm laser for the structured light projection (Photoneo, 2018). We also record the interaction with a Kinect RGB-D camera that uses laser diodes with peak intensity at 850 nm for time-of-flight measurement (Naeemabadi et al., 2018) and separate sensors for the visible part of the spectrum and for near-infrared. To calibrate all cameras internally and externally, we developed a calibration target (Figure 2). The pattern is a 4 by 11 asymmetric circle grid with 20.0 mm spacing and 15.0 mm circle diameter. The pattern was cut by laser from a 70 μm thick matte black PVC film and attached to a 4.0 mm thick unpolished aluminum alloy sheet. We warmed the calibration target with a heating element so that the temperature difference with room temperature was approximately 10°C. The uncoated aluminum circles reflect the surrounding environment in the wavelengths recorded by the devices, whereas the PVC-coated surface is less reflective. The calibration target enables us to utilize the camera calibration implementation in OpenCV (https://opencv.org/). We calibrated the camera internal parameters and the camera system externally, so that we obtained a rotation and translation relative to the 3D scanner for each camera.

**FIGURE 3:**
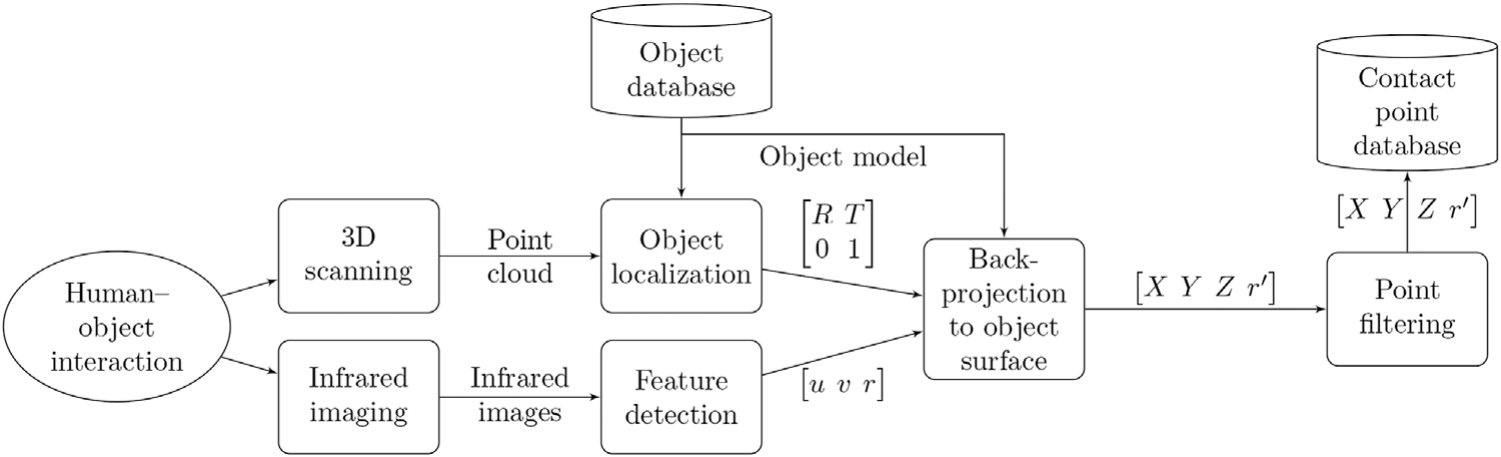
The stages of measuring contact points. After human-object interaction, we record infrared images of the scene and a 3D point cloud. We use the point cloud to localize the object in the scene accurately and a computer-vision algorithm detects the contact points in the infrared images. Next, we project the contact points to the surface of the localized object model, filter, and store the point coordinates and radii.

Data acquisition takes place during and after the interaction. We record the scene with the infrared cameras and one RGB-D camera during the interaction and a short period after the interaction has ended. At the end of the interaction, the subject places the object on the table covered by the cameras and pulls their hand out of the line of sight between the object and the cameras. Then, the recording ends and we scan the scene with the 3D scanner. Figure 3 illustrates the process flow. Here, we describe the process for a single object, but the method allows for efficient data gathering from multiple objects consecutively. The experiment supervisor needs to take care to place each object in the desired starting location without altering its surface temperature, for example, by wearing insulating gloves or using room-temperature tongs.

To detect the contact points in the infrared images recorded after the interaction, we used the simple blob detection algorithm implemented in OpenCV. The algorithm detects the regions that differ in temperature from the ambient temperature, and outputs the center point coordinates u and v, and radius r of each region. The camera parameters used and the scene configuration determine the required parameters for the algorithm.

For localization, we used the proprietary Photoneo Localization SDK (Photoneo s.r.o., Bratislava, Slovakia). The localization algorithm uses a set of features calculated from a predefined 3D model of the object and searches for matching features in the recorded point cloud. As a result of the localization, we obtain the object rotation and transformation matrices, R and T, relative to the 3D scanner.

To map the contact points on the object surface, we project the contact points from the image coordinates back to the 3D scene, where the rays projected from the camera intersect with the surface of the object 3D model. To solve the visibility of the points and self-occlusions within the object, we use OpenGL (Khronos Group, Beaverton, OR, United States) to obtain the Z-buffer of the object model from each infrared camera location. We transform the object model from the 3D scanner reference frame to the IR camera reference frame and render it. Thus, the resulting Z-buffer provides the Z-depth of the key points in the infrared image. Points outside the object boundary are discarded. The key point coordinates are then transformed to the object reference frame.

As the same contact point may be visible in multiple camera images, we filter the set of contact points. As a measure of point reliability, we use the angle between the camera optical axis and the surface normal at the contact point, because the imperfections in key point detection, calibration, and localization cause an error in projection that increases with the incident angle. Out of the key points from different cameras that are within a 9 mm distance of each other, we retain the point with the smallest incident angle. Finally, we store the remaining key point coordinates X, Y and Z, and radii r’, in millimeters in object coordinates, within the database.

We followed the guidelines given by the Finnish Advisory Board on Research Integrity and the University of Helsinki Ethical Review Board in the Humanities and Social and Behavioural Sciences.

## Results

**FIGURE 4:**
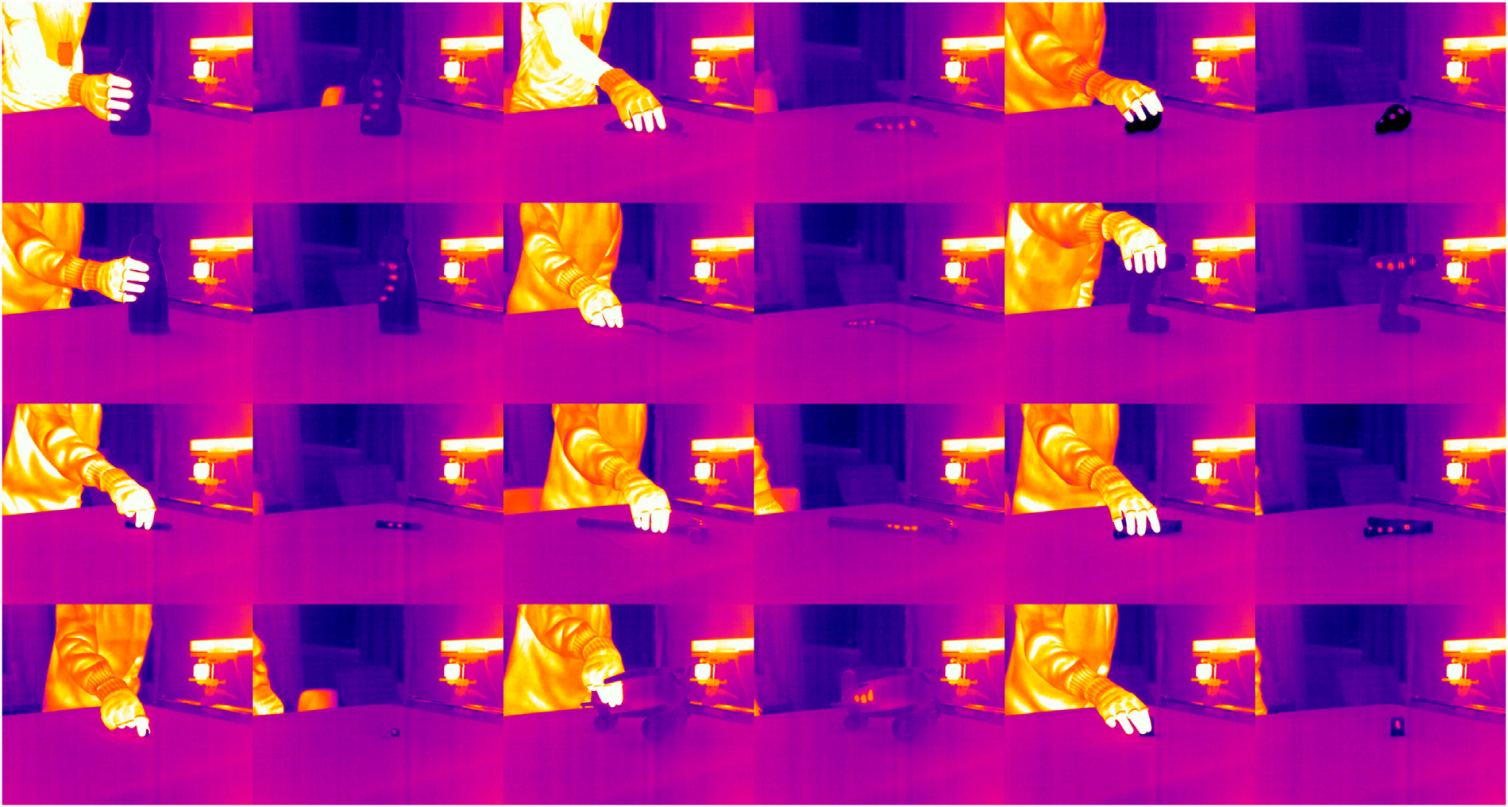
Twelve sample infrared image pairs recorded during the grasping act and after the interaction.

We recorded one grasping event for each of the 77 objects in the YCB object set (Calli et al., 2015; Calli et al., 2017) for which there was a matching 3D model available. Author JHH grasped each object with his right hand using a natural grasp suitable for lifting the object (Figure 4), lifted the object from the table to 10–20 cm height, and set it back down. The grasp type or duration was not restricted; contact duration was often less than 2 s. To limit the skin–object contact areas to the distal phalanx of each finger, the grasper wore an insulating fingerless glove.

Localization succeeded for 67 out of the 77 objects. Localization was unsuccessful possibly due to partially deformed objects (004_sugar_box and 061_foam_brick), non-rigidity of the object (059_chain and 063-a_marbles), and highly reflective surfaces (038_padlock). The localization result of 019_pitcher_base was 180° rotated and thus classified as a failure.

**FIGURE 5:**
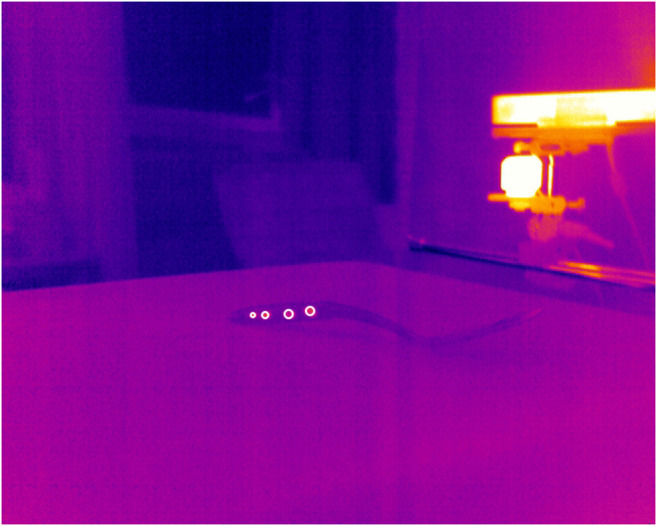
Detected key points illustrated as white circles in the infrared image of the object 033_spatula.

**FIGURE 6:**
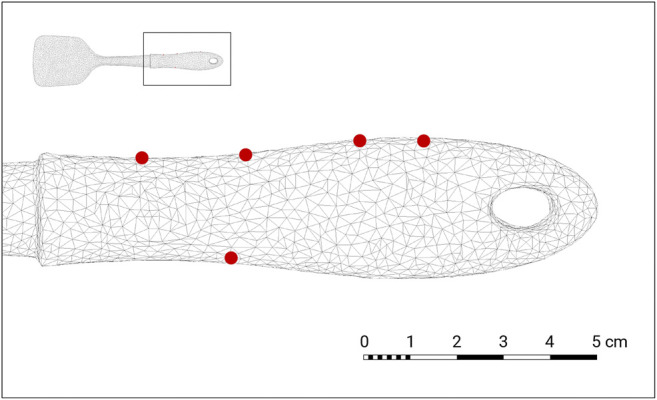
Result contact points visualized as red dots over the wireframe model of the object 033_spatula.

**FIGURE 7:**
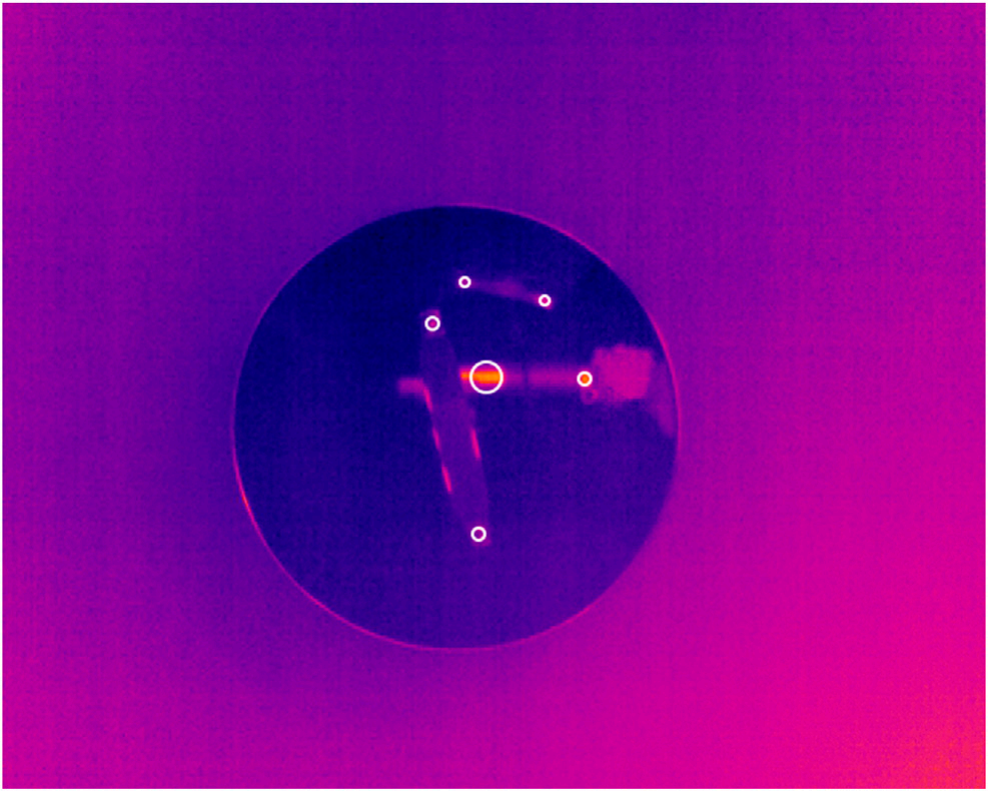
Highly reflective surfaces, such as the glass dome in 028_skillet_lid depicted here, caused the majority of false positive detections.

Determining the ground truth for measuring the performance of the method proved challenging, as there is no automated method that could provide the ground truth. We determined the success of the contact point detection by visually comparing the detected contact points to the IR and RGB camera frames from the grasping event. Thus, our “ground truth” is prone to human error, but we provide the grasping event recordings along with our code^1^, so others may confirm our results. Detection of some contact points succeeded in 66 out of the 67 localized objects. Figures 5, 6 show examples of the detections. Only the highly reflective object 042_adjustable_wrench did not yield any true positive detections. For 62 objects, the method detected all contact points successfully. False positive detections occurred in 16 objects, mainly due to reflections in high-reflectance objects such as 028_skillet_lid, as evident in Figure 7. The insulating glove covered the second phalanx of the grasper’s fingers only partially, which resulted in two contact points from the same finger in three cases. Some contact points went undetected because the surface was not covered by the cameras, such as the underside of the object 029_plate. We computed precision and recall for all finger contacts and used visual inspection of the recordings as the ground truth. For the 67 localized objects, the precision of the system was 0.90 whereas recall was 0.95.

## Discussion

In this article, we have described a novel method for measuring contact points in human–object interaction and presented the first test measurement results. We measured grasping with the YCB object set, which includes everyday objects with different sizes and materials. For most of the objects, we were able to measure contact points successfully. For the 22 plastic toy objects in groups 065, 072, and 073 precision was 0.97 and recall 1.00, which indicates that in applications, where the experimenter can control the object material, the method meets demanding requirements. Highly reflective objects proved difficult for both the 3D scanner and the infrared cameras to measure.

The spatial accuracy of the process is challenging to measure, because the error depends on the properties of the environment, the object, the grasping hand, and grasp type. We could measure the error using an ideal object with a known geometry and temperature difference as a target, such as our calibration target. However, such a measure, likely in fractions of a millimeter, would be highly misleading as it discounts the major sources of error. Thermal conduction over time within the object should be taken into account when considering the spatial accuracy. We are developing a method to measure the spatial accuracy of the entire process; from our experience, we expect the root mean square error to be within a few millimeters with our hardware and software configuration.

The method is efficient and accurate for measuring contact points. For some applications, a more realistic model of the contact area might be more desirable than only the contact area centers and radii that our method currently measures. For example, in robotics, a patch contact model improves grasping compared with a point model (Charusta et al., 2012). Our method is also extendable to measuring the contact areas, by detecting the contact areas in the infrared camera images and mapping them to the model surface. For contact area measurement, the temperature gradient caused by thermal conduction within the object must included in the model, as the contact areas cool down over time after the contact. This could be achieved by tracking the object and recording the contact duration and surface area temperature over time. However, as the fingers of the grasper occlude the contact area, the exact duration of contact is challenging to measure from unaltered objects. Including the object surface’s material properties and three-dimensional object structure in the model would be useful.

Another improvement we are developing is including a hand and finger tracking method. Tracking would allow us to identify fingers, collect motion trajectory data, and determine the grasp type, which is valuable in some applications (such as grasp planning, e.g., in GraspIt! (Miller and Allen, 2004). To increase the usefulness and improve the performance of the method, prior knowledge about the grasp type and the number of contact points used should be included in data processing, as different grasp types produce different skin-object contact areas. In addition to the contact points and patches, some applications may require additional data. Using, e.g., finite element models for the deformation of fingers might provide useful. Furthermore, measuring the force distribution and friction between the contact surfaces would be very useful in robotics applications. Combining the models for thermal conduction and finger deformation would allow us to approximate the grasp forces. The weights and dimensions of the objects, which might also be useful, are listed in the YCB dataset description (Calli et al., 2015).

In automation and robotics, the method enables the recording of grasping demonstrations with known objects in applications such as warehouse or assembly line automation, if the number of target objects makes this feasible. The method also enables the collection of the vast datasets required for training machine-learning models and the subsequent development of robots that are able to operate in unstructured environments such as households. For example, action-specific contact point and point cloud data recorded from a large number of objects enables the training of a deep neural network to predict contact points in novel objects, a task where manually annotated images are still used (Chu et al., 2018).

## Data Availability

The original contributions presented in the study are included in the article/Supplementary Material, further inquiries can be directed to the corresponding author. Infrared and RGB recordings of grasping YCB objects are available at doi.org/10.5281/zenodo.5783717.

## Footnotes

1
https://github.com/jussihh/graspsense

## Supplementary Material

The Supplementary Material for this article can be found online at: https://www.frontiersin.org/articles/10.3389/frobt.2021.800131/full#supplementary-material Supplementary Material: Contains sample data recorded from the YCB object set with the method described in this article. See the included readme.txt for detailed description of the data.
